# Adherence to mediterranean diet and the risk of differentiated thyroid cancer in a European cohort: The EPIC study

**DOI:** 10.3389/fnut.2022.982369

**Published:** 2022-09-02

**Authors:** Fjorida Llaha, Valerie Cayssials, Marta Farràs, Antonio Agudo, Maria Sandström, Anne Kirstine Eriksen, Anne Tjønneland, Marie-Christine Boutron-Ruault, Nasser Laouali, Thérèse Truong, Charlotte Le Cornet, Verena Katzke, Matthias Schulze, Domenico Palli, Vittorio Krogh, Simona Signoriello, Rosario Tumino, Fulvio Ricceri, Guri Skeie, Torill Miriam Enget Jensen, Sairah Lai Fa Chen, Cristina Lasheras, Miguel Rodriguez-Barranco, Pilar Amiano, José María Huerta, Marcela Guevara, Martin Almquist, Lena Maria Nilson, Joakim Hennings, Keren Papier, Alicia Heath, Elisabete Weiderpass, Sabina Rinaldi, Raul Zamora-Ros

**Affiliations:** ^1^Unit of Nutrition and Cancer, Epidemiology Research Program, Catalan Institute of Oncology, Bellvitge Biomedical Research Institute (IDIBELL), Barcelona, Spain; ^2^Department of Veterinary Public Health, Faculty of Veterinary, University of the Republic, Montevideo, Uruguay; ^3^Department of Quantitative Methods, Faculty of Medicine, University of the Republic, Montevideo, Uruguay; ^4^Department of Radiation Sciences, Oncology, Umeå University, Umeå, Sweden; ^5^Unit of Diet, Genes and Environment, Danish Cancer Society Research Center, Copenhagen, Denmark; ^6^University Paris-Saclay, University of Versailles Saint-Quentin-en-Yvelines (UVSQ), Institut National de la Santé et de la Recherche Médicale (INSERM), Gustave Roussy, Centre de Recherche en Epidémiologie et Santé des Populations (CESP), Team “Exposome and Heredity”, Villejuif, France; ^7^Division of Cancer Epidemiology, German Cancer Research Center (DKFZ), Heidelberg, Germany; ^8^Department of Molecular Epidemiology, German Institute of Human Nutrition Potsdam-Rehbruecke, Nuthetal, Germany; ^9^Institute of Nutritional Science, University of Potsdam, Nuthetal, Germany; ^10^Cancer Risk Factors and Life-Style Epidemiology Unit, Institute for Cancer Research, Prevention and Clinical Network - Institute for the Study and Prevention of Cancer, Florence, Italy; ^11^Epidemiology and Prevention Unit, Fondazione Istituto Nazionale dei Tumori (IRCCS), Milan, Italy; ^12^Dipartimento di Salute Mentale e Fisica e Medicina Preventiva, Vanvitelli University, Naples, Italy; ^13^Hyblean Association for Epidemiological Research (AIRE -ONLUS), Ragusa, Italy; ^14^Department of Clinical and Biological Sciences, University of Turin, Turin, Italy; ^15^Department of Community Medicine, Faculty of Health Sciences, University of Tromsø (UiT) - The Arctic University of Norway, Tromsø, Norway; ^16^Department of Functional Biology. Medical School. University of Oviedo, Oviedo, Spain; ^17^Escuela Andaluza de Salud Pública, Granada, Spain; ^18^Instituto de Investigación Biosanitaria Granada (ibs.GRANADA), Granada, Spain; ^19^Centro de Investigación Biomédica en Red de Epidemiología y Salud Pública (CIBERESP), Madrid, Spain; ^20^Ministry of Health of the Basque Government, Sub-Directorate for Public Health and Addictions of Gipuzkoa, San Sebastian, Spain; ^21^Public Health Division of Gipuzkoa, BioDonostia Research Institute, San Sebastian, Spain; ^22^Department of Epidemiology, Murcia Regional Health Council, Biomedical Research Institute of Murcia (IMIB)-Arrixaca, Murcia, Spain; ^23^Navarra Public Health Institute, Pamplona, Spain; ^24^Navarra Institute for Health Research (IdiSNA), Pamplona, Spain; ^25^Department of Surgery, Skåne University Hospital, Lund University, Lund, Sweden; ^26^Department of Epidemiology and Global Health, Umeå University, Umeå, Sweden; ^27^Department of Surgical and Perioperative Sciences/Surgery, Umeå University, Umeå, Sweden; ^28^Cancer Epidemiology Unit, Nuffield Department of Population Health, University of Oxford, Oxford, United Kingdom; ^29^Department of Epidemiology and Biostatistics, School of Public Health, Imperial College London, London, United Kingdom; ^30^International Agency for Research on Cancer – World Health Organization (IARC-WHO), Lyon, France

**Keywords:** thyroid cancer (TC), Mediterranean diet (MD), meat, intake, EPIC study, cohort

## Abstract

**Background:**

The Mediterranean diet (MD) has been proposed as a healthy diet with a potential to lower the incidence of several types of cancer, but there is no data regarding thyroid cancer (TC). We investigated the association between MD adherence, and its components, and the differentiated TC risk within the European Prospective Investigation into Cancer and Nutrition (EPIC) cohort.

**Methods:**

Over 450,000 men and women from nine European countries were followed up for a mean of 14.1 years, during which 712 differentiated TC cases were identified. Adherence to MD was estimated using the relative MD (rMED) score, an 18-point scale including alcohol, and the adapted rMED (arMED) score, a 16-point scale excluding alcohol. Hazard ratios (HRs) and 95% confidence intervals (CIs) were estimated using Cox regression models adjusted for potential confounding factors.

**Results:**

Adherence to the arMED score was not associated with the risk of differentiated TC (HR_high vs. low adherence_ = 0.94, 95% CI: 0.70–1.25; *p*-trend 0.27), while a suggestive, but non-statistically significant inverse relationship was observed with rMED (HR_high vs. low adherence_ = 0.88, 95% CI: 0.68–1.14; *p*-trend 0.17). Low meat (HR_low vs. high meat intake_ = 0.81, 95% CI: 0.67–0.99; *p*-trend = 0.04) and moderate alcohol (HR_moderate vs. non−moderate intake_ = 0.88, 95% CI: 0.75–1.03) intake were related with lower differentiated TC risk.

**Conclusions:**

Our study shows that a high adherence to MD is not strongly related to differentiated TC risk, although further research is required to confirm the impact of MD and, especially, meat intake in TC risk.

## Introduction

Thyroid cancer (TC) represents the most common endocrine malignancy worldwide (1). Lastly, the TC incidence has gradually increased, in part driven by overdiagnosis due to the use of ultrasound examinations and increased medical surveillance, leading to higher TC prevalence in high-income countries (2). The transformation of thyroid follicular cells may result in differentiated or undifferentiated TC. Differentiated TC, including papillary and follicular carcinoma, represents more than 90% of all TC cases (3). Poorly differentiated and anaplastic thyroid carcinomas are rare but more aggressive tumor types (3). Exposure to ionizing radiation, particularly during childhood (4), previous history of benign thyroid hyperplasia (5), and overweight/obesity (6, 7) are the mostwell-established risk factors for TC.

The Mediterranean diet (MD) is characterized by a high consumption of fruits, vegetables, complex carbohydrates and fish, a low amount of meat and dairy products and a daily glass of wine (8). In this dietary pattern, olive oil is the main source of fats (9). There is evidence that relates high adherence to MD with lower risk of cancer incidence and mortality (e.g., breast, colorectal, head and neck, respiratory, gastric, liver and bladder) (10, 11), obesity (12), and type 2 diabetes (13). Convincing evidence is consistently showing a positive moderate association for overweight and obesity (6, 7), and type 2 diabetes (14, 15) with TC incidence. MD is rich in polyphenols, fibers, phytosterols, monounsaturated and polyunsaturated fatty acids, which are probably the main drivers of the protection of MD against cancer (16). The potential underlying mechanisms of action involve anti-oxidative and anti-inflammatory effects, reduction of tumor cell growth, increase of chemoprotective effects, and inhibition of tumor development (16). Several dietary factors of MD have been suggested to play a role in TC etiology, but the results are inconclusive (17, 18). Previous studies investigating TC have mainly focused on separate food items and only few on dietary patterns (17, 18). Dietary pattern analysis examines the overall effects of diet and could be a better approach to investigate the role of diet in chronic diseases (19).

To our knowledge, there are no studies on the relationship between MD adherence and TC risk. Therefore, in the current study we aimed to investigate the association between MD adherence and the risk of differentiated TC within the European Prospective Investigation into Cancer and Nutrition (EPIC) study.

## Materials and methods

### Subjects and study design

EPIC is a large prospective cohort study designed to investigate the relationship between diet, lifestyle, environmental factors, and cancer. The full methods and study design have been described previously (20). In brief, 521,324 participants, mostly aged between 35 and 70 years, were recruited between 1992 and 2000 in 23 centers from 10 Western European countries. All participants provided written informed consent, and the study was approved by the local ethics committees in the participating countries and the ethical review board of the International Agency for Research on Cancer (IARC). We excluded participants with prevalent cancer other than non-melanoma skin cancer at baseline or with missing information on date of diagnosis or censoring data, missing dietary and lifestyle information (did not complete the questionnaires), had extreme energy intake and/or expenditure (participants in the top or bottom 1% of the distribution of the ratio of total energy intake to energy requirement) and participants from Greece (data not provided for the current study) (Supplementary Figure 1).

### Dietary and lifestyle ascertainment

Dietary information was collected at enrollment, using country-specific dietary questionnaires (20). Total energy intake was estimated by using the standardized EPIC Nutrient Database (21). At baseline, information on socio-demographic characteristics, tobacco consumption, physical activity, reproductive history, use of oral contraceptives and hormone replacement therapy, and medical history were self-reported using standardized lifestyle questionnaires (20). Anthropometry (weight and height) was measured at recruitment by trained personnel, except for Oxford (United Kingdom), Norway, and France, where measurements were self-reported.

The adherence to MD was measured using the adapted relative MD score (arMED), a version of the relative MD (rMED) (22) based on the original MD score by Trichopoulou et al. (23), excluding alcohol. The arMED incorporates 8 selected components of MD and is a 16-point scale. Each component was calculated as a function of energy density (g/2,000 kcal per day) and then divided into cohort-wide tertiles of intakes (except for olive oil). For five of the six components that positively reflect MD: fruits (including nuts and seeds), vegetables (excluding potatoes), legumes, fish (including seafood), and cereal products, a score of 0-1-2 was assigned to the lowest to highest intake tertiles, respectively. The score was inverted (2-1-0 assigned to the intake tertiles) for the two components that negatively reflect MD: total meat (red meat, processed meat, poultry, game, and offal) and dairy products. The score for olive oil was adapted for non-Mediterranean countries owing to their low consumption, by assigning 0 to non-consumers, 1 for subjects below the median intake and 2 for subjects equal to or above this median. The arMED score was further classified into low (0–5 points), medium (6–9 points) or high (10–16 points) adherence levels, as previously categorized in the EPIC study (10).

In a previous EPIC study, moderate alcohol intake was inversely associated with differentiated TC risk (24). Therefore, the rMED score (22), including alcohol, was also computed. The rMED incorporates the same previous 8 components plus alcohol and is an 18-point scale. Alcohol in the rMED score was scored dichotomously assigning 2 points for moderate consumption (sex-specific cut off points: 5–25 g per day for women and 10–50 g per day for men) and 0 points for intakes outside this range. The rMED score was further classified into low (0–6 points), medium (7–10 points) or high (11–18 points) adherence levels, as previously categorized in the EPIC study (22, 25).

### Follow-up and case assessment

Incident cancer cases were identified through population cancer registries in all countries except France Germany, and Naples (Italy) where cases were identified through active follow-up, directly from the participants and confirmed by a combination of methods, including health insurance records, and cancer and pathology registries. Vital status was obtained from mortality registries at the regional or national level. Complete follow-up censoring dates ranged from December 2010 to December 2014, depending on the study center. In this TC study (code C73 according to the International Classification of Diseases, 10th Revision), only first primary differentiated TC cases were included, and therefore, 52 undifferentiated TC (such as medullary, anaplastic, lymphoma, and other morphologies) were excluded (Supplementary Figure 1). Finally, 712 first primary incident differentiated TC cases were considered: 573 papillary TCs, 108 follicular TCs, and 31 not otherwise specified (NOS) TCs, most likely to be papillary TCs. Data on the stage of differentiated TC at diagnosis were collected from each center where possible. A total of 468 cases (65.7%) had tumor-node-metastasis staging score information, of which 371 were classified as low-risk tumors (T1–T2) and 97 were classified as high-risk tumors (T3–T4).

### Statistical analysis

Cox proportional hazard models were used to estimate hazard ratios (HR) and 95% confidence intervals (CI) between adherence to the MD measured by arMED and rMED score, its individual components and differentiated TC risk. Age was the primary time variable in all models. Entry time was age at recruitment and exit time was age at first diagnosis (cases), death, or censoring date (loss or end of follow-up), whichever occurred first. Both arMED and rMED scores were computed as categorical variables (low, medium, and high) and as continuous variables (per 1-unit). Trend tests were obtained by scoring the arMED or rMED categories in a continuous scale from 1 to 3. The basic model for the association between arMED and differentiated TC was stratified by sex, age at recruitment (1-year interval), study center, and adjusted for total energy intake (kcal/day). Variables associated with TC in previous EPIC studies (24, 26–28) were a-priori selected as potential confounders. Thus, the most-adjusted model was additionally adjusted for body mass index (BMI, kg/m^2^), smoking status, alcohol (g/day), education level and physical activity (according to the Cambridge Physical Activity Index) (29). In women the model was also adjusted for menopausal status and type, ever use of oral contraceptives, and history of infertility problems. Results from the two models were almost identical, therefore, the most-adjusted model was selected for presentation. Similar models were applied for the rMED score, without alcohol consumption (g/day) as adjustment variable. All models met the proportional hazard assumption, tested using the Schoenfeld goodness-of-fit test. In addition, we estimated the associations between individual components of MD and TC risk. Each component was evaluated as a categorical variable (tertile points assigned for the arMED/rMED score calculation), except for alcohol (moderate vs. non-moderate consumption). Interactions on the multiplicative scale with sex, smoking status (never, former, and current smokers), alcohol (low, moderate, and high), BMI (<25 and ≥25 kg/m^2^), were examined using the likelihood ratio test.

Separate analyses were performed for differentiated TC subtypes: follicular and papillary tumors, and disease stage: low-risk (T1–T2) and high-risk (T3–T4) tumors. Heterogeneity of risk between TC subtypes was assessed with the Wald test. Separate models were also computed to check the variability between countries with a high and low TC incidence. EPIC countries with TC incidence rates of >5/10,000 in women (i.e., France, Germany, Italy, and Spain) were considered to have a high TC incidence. Moreover, separate models were conducted only in women, because of the small proportion of men with TC (10.4%). Finally, we conducted separate analyses by geographical regions: South (Spain, Italy, France), Central (UK, Germany, and the Netherlands) and North Europe (Denmark, Norway, and Sweden) because dietary habits can differ between European regions (30). Sensitivity analyses were performed by repeating the models after the exclusion of differentiated TC cases diagnosed during the first 2 years of follow-up, since participants may have changed their diets in the pre-diagnostic period. For all analyses, *p*-values < 0.05 were considered statistically significant. Statistical analyses were conducted using R 3.2.1 software.

## Results

In the current analysis of 450,064 EPIC participants (70.8% women), 712 were diagnosed with differentiated TC (89.6% women) (Supplementary Figure 1). The mean arMED score was 7.8 (3.0) ranging between 4.6 (in Sweden) and 10.7 points (in Spain) (Table 1). Participants with high arMED score were more likely to be women, younger, never smoker, physically inactive/moderate inactive, and to consume less alcohol and slightly less total energy at recruitment, compared to those with a lower arMED score (Table 2). Women with high arMED score compared to those with low, tended to be premenopausal.

**Table 1:** Description of the EPIC study by country and by adapted relative Mediterranean diet score (arMED).

			**Differentiated thyroid cancer cases (** ***n*** **)**				**arMED score**		**arMED score (%)**		
**Country**	***N***	**Women (%)**	**Overall**	**Papillary**	**Follicular**	**NOS**	**Mean**	**SD**	**Low (0–5)**	**Medium (6–9)**	**High (10–16)**
Denmark	55,005	52.2	39	28	11	0	6.0	2.5	44.5	46.4	9.1
France	67,391	100	248	227	19	2	8.9	2.4	7.5	51.7	40.8
Germany	48,551	56.4	82	58	21	3	6.6	2.2	31.6	58.2	10.2
Italy	44,543	68.5	127	97	19	11	10.4	2.1	0.9	33.9	65.2
Norway	33,972	100	36	31	4	1	7.8	2.1	13.6	65.8	20.6
Spain	39,984	62.1	80	66	13	1	10.7	2.2	1.3	26.2	72.5
Sweden	48,666	54.2	39	25	7	7	4.6	2.1	69.0	29.4	1.5
Netherlands	36,537	73.7	17	12	4	1	5.8	2.1	46.9	48.3	4.9
UK	75,415	69.7	44	29	10	5	8.7	2.4	10.1	51.6	38.2
Total	450,064	70.8	712	573	108	31	7.8	3.0	24.2	46.1	29.7

NOS: not otherwise specified; SD standard deviation.

**Table 2:** Baseline characteristics of included participants from the EPIC study according to the adapted relative Mediterranean diet score (arMED).

**Characteristics**	**All**	**arMED score**		
		**Low (0-5)**	**Medium (6–9)**	**High (10–16)**
*N*	450,064	108,791	207,470	133,803
**Sex, %**				
Women	70.8	53.6	74.7	78.9
Men	29.2	46.4	25.3	21.3
Age, years [mean (SD)]	51.1 (9.8)	51.9 (10.0)	51.5 (9.5)	49.9 (9.7)
Total energy, kcal/day [mean (SD)]	2,077 (619)	2,176 (654)	2,039 (601)	2,054 (607)
Alcohol, g/day [median (IQR)]	5.5 (0.9-15.2)	6.0 (1.3-16.8)	5.7 (1.1-15.2)	4.9 (0.5-13.5)
**BMI, %**
<25 kg/m^2^	53.3	48.3	55.1	54.4
25 to <30 kg/m^2^	34.4	38.6	33.4	32.6
≥30 kg/m^2^	12.4	13.1	11.5	13.0
**Smoking status (%)**				
Never	48.7	41.1	48.8	54.8
Former	27.3	27.5	28.1	25.7
Current	22.2	30.1	20.9	17.6
Unknown	1.9	1.2	0.2	1.9
**Physical activity (%)**				
Inactive or moderately inactive	52.9	49.3	51.2	58.3
Active or moderately active	45.2	47.6	46.6	41.0
Unknown	2.0	3.2	2.2	0.7
**Education level (%)**				
Primary or lower	28.1	31.0	24.3	31.7
Secondary or higher	68.1	67.3	71.3	63.8
Unknown	3.8	1.7	4.4	4.5
**Menopausal status and type**^**a**^ **(%)**				
Premenopausal	34.7	29.5	33.2	39.9
Perimenopausal	19.8	20.6	20.6	17.9
Postmenopausal	42.8	47.6	43.6	38.9
Surgical menopause	2.8	2.3	2.6	3.3
**Ever use of contraceptive pill**^**a**^ **(%)**				
No	37.9	33.4	36.5	42.5
Yes	59.4	58.6	61.5	56.9
Unknown	2.6	7.9	2.0	0.6
**Infertility problems**^**a**^ **(%)**				
No	62.3	37.1	60.9	78.4
Yes	3.1	1.3	2.9	4.2
Unknown	34.6	61.6	36.2	17.4

arMED, adapted relative Mediterranean diet; BMI, body mass index; IQR, interquartile range; SD, standard deviation.

a Only in women (*n* = 318,647).

We found no association between arMED score (excluding the alcohol component) and the risk of overall differentiated TC in the fully-adjusted model (HR_high vs. low adherence_ = 0.94, 95% CI: 0.70–1.25; *p*-trend = 0.27) (Table 3). No differences were observed in associations by TC subtype (*p*-value for heterogeneity = 0.82). No interactions were found for sex, smoking status, BMI, and alcohol intake. No statistically significant differences were observed in the associations between arMED score and differentiated TC risk by tumor stage, country-incidence rate, and European region (Supplementary Table 1). Similar non-statistically significant results were observed in women only and in the sensitivity analysis excluding the TC cases diagnosed within the first 2-years of follow-up (Supplementary Table 1).

**Table 3:** Hazard ratios (95% Confidence Intervals) for the associations between relative Mediterranean diet score (rMED), adapted rMED (arMED) and differentiated thyroid cancer (TC) risk in the EPIC study.

	***N***	**Overall differentiated TC**	**Papillary TC**	**Follicular TC**	**p for heterogeneity**
Cases (*n*)		712	573	108	
arMED score (adjusted for alcohol)					0.82
Low (0–5)	108,791	1.00 (ref)	1.00 (ref)	1.00 (ref)	
Medium (6–9)	207,470	1.13 (0.88–1.45)	1.14 (0.85–1.52)	1.26 (0.70–2.27)	
High (10–16)	133,803	0.94 (0.70–1.25)	0.96 (0.70–1.33)	0.99 (0.48–2.03)	
*P*-trend		0.27	0.38	0.83	
Continuous (per unit)	450,064	0.98 (0.95–1.02)	0.99 (0.95–1.03)	0.99 (0.91–1.08)	
rMED score (not adjusted for alcohol)					0.58
Low (0–6)	121,208	1.00 (ref)	1.00 (ref)	1.00 (ref)	
Medium (7–10)	201,933	1.05 (0.84–1.32)	1.10 (0.85–1.42)	0.88 (0.51–1.51)	
High (11–18)	126,923	0.88 (0.68–1.14)	0.87 (0.65–1.17)	0.97 (0.51–1.84)	
*P*-trend		0.17	0.14	0.97	
Continuous (per unit)	450,064	0.98 (0.95–1.01)	0.98 (0.94–1.01)	0.98 (0.91–1.07)	

Cox models were stratified by sex, age at recruitment, study center, and adjusted for total energy intake (kcal/day, continuous), body mass index (kg/m^2^, continuous), smoking status, alcohol (g/day, continuous, when applied), education level, and physical activity. In addition, in women, they were further adjusted for menopausal status and type, ever use of oral contraceptives, and history of infertility problems.

A non-statistically significant inverse relationship between rMED score (including the alcohol component) and overall differentiated TC (HR_high vs. low adherence_ = 0.88, 95% CI: 0.68–1.14; *p*-trend = 0.17), especially against papillary TC risk (HR_high vs. low adherence_ = 0.87, 95% CI: 0.65–1.17; *p*-trend = 0.14) was observed (Table 3). In the analysis of each component of MD, we found an inverse association between low meat intake and differentiated TC risk (HR_low vs. high adherence_ = 0.81, 95% CI: 0.67–0.99) (Table 4). The HR for moderate vs. non-moderate alcohol intake was 0.88 (95% CI 0.72–1.03). The other components were not related to differentiated TC risk.

**Table 4:** Hazard ratios (95% Confidence Intervals) of the association between each component of Mediterranean diet (MD) and differentiated thyroid cancer risk in the EPIC study.

**rMED components** **(g/day per 2,000 kcal)**	**Median (33–67th percentiles)**	**0 point at rMED**	**1 point at rMED**	**2 points at rMED**	***P*-trend**
Vegetables	167.7 (125.9–221.1)	1.00 (ref)	0.99 (0.81–1.21)	0.89 (0.71–1.11)	0.26
Fruits	193.1 (135.3–265.0)	1.00 (ref)	1.08 (0.88–1.32)	1.07 (0.86–1.31	0.60
Legumes	4.8 (0.8–11.3)	1.00 (ref)	1.01 (0.82–1.26)	0.94 (0.74–1.19)	0.57
Cereals	170.4 (138.8–204.3)	1.00 (ref)	0.98 (0.81–1.18)	0.92 (0.75–1.12)	0.39
Olive oil	0.0 (0.0–0.8)	1.00 (ref)	1.08 (0.85–1.39)	1.19 (0.94–1.50)	0.14
Fish	17.9 (10.2–27.7)	1.00 (ref)	1.10 (0.89–1.36)	1.20 (0.95–1.51)	0.13
Meat	94.1 (74.5–114.5)	1.00 (ref)	0.95 (0.79–1.13)	0.81 (0.67–0.99)	0.04
Dairy	286.0 (205.9–386.0)	1.00 (ref)	0.93 (0.77–1.12)	0.86 (0.71–1.05)	0.15
Alcohol (categorical)	5.6 (2.1–10.9)	1.00 (ref)		0.88 (0.75–1.03)	

rMED, relative Mediterranean diet score. Each component was calculated as a function of energy density (g/2,000 kcal per day) and then divided into tertiles of intakes (except for olive oil and alcohol). In the rMED score, for five of the six components that positively reflect Mediterranean diet: fruits (including nuts and seeds), vegetables (excluding potatoes), legumes, fish (including seafood), and cereals, points of 0-1-2 were assigned to the intake tertiles. For olive oil, 0 was assigned to non-consumers, 1 for subjects below the median intake and 2 for subjects equal or above this median. For meat and dairy products, which negatively reflect Mediterranean diet, 2-1-0 points were assigned to the first, second and third intake tertiles, respectively. Alcohol was scored dichotomously assigning 2 points for moderate consumption (sex-specific cut off points: 5–25 g per day for women and 10–50 g per day for men) and 0 points for intakes outside this range. Cox models were stratified by sex, age at recruitment, study center, and adjusted for total energy intake (kcal/day, continuous), body mass index (kg/m^2^, continuous), smoking status, alcohol (except for the alcohol model), education level, physical activity. In addition, in women they were further adjusted for menopausal status and type, ever use of oral contraceptives, and history of infertility problems.

## Discussion

Adherence to MD, measured by arMED score (without the alcohol component) was not associated with the risk of differentiated TC in this large European prospective cohort study (*n* = 450,064) with a long follow-up (mean = 14.1 years), and a relatively high number of cases (*n* = 712). The results were also non-statistically significant in all sub-analyses. However, there was a statistically non-significant inverse relationship with rMED (including the alcohol component), probably driven by the inverse trend with alcohol intake and the positive association with meat intake.

In our longitudinal study, we did not find a clear association of differentiated TC risk with MD adherence. Whereas, in an US population-based case-control study, a tendency for an inverse association between a dietary pattern high in fruits and vegetables and risks of both overall and papillary TC were observed (31). Similarly, a traditional Polynesian dietary pattern characterized by a high consumption of fish, seafood and fruits, and low consumption of meat was inversely related, but was not statistically significant, with overall and papillary TC risk (32). In a Greek case-control study, inverse associations were found between the risk of overall and papillary TC and three dietary patterns rich in fresh fruit, raw vegetables, and mixed raw vegetables and fruits. Contrarily, a dietary pattern rich in fish and cooked vegetables, which is a dietary habit of Mediterranean populations, showed a higher risk of follicular TC (33). In a cross-sectional study, a high adherence to MD correlated with lower circulating levels of triiodothyronine (T3) and thyroxine (T4), but not with thyroid-stimulating hormone (TSH) (34). However, associations of TC with hypo- or hyperthyroidism and thyrotoxicosis are weaker and less consistent. High concentrations of free T4, TSH and the T4/T3 ratio were related to a higher differentiated TC risk in a small Canadian case-control study (35). Nevertheless, in a previous EPIC analysis, only low levels of TSH and high levels of thyroglobulin were associated with a higher differentiated TC risk, but not plasma concentrations of either T3 or T4 (36).

Except meat and a suggestive association for alcohol, none of the other components presumed to fit MD were related to differentiated TC risk in our analysis. In previous EPIC analyses, similar null results were observed with the consumption of fruit and vegetables (37). Likewise, a meta-analysis using 19 case-control studies found no association with the intake of fruit and vegetables including cruciferous vegetables, which have been studied in more detail due to their content of goitrogens (38). Fish is a rich natural source of iodine which is essential for thyroid function. A meta-analysis of six case-control studies suggested that consumption of fish may decrease the risk of TC in iodine deficient areas, but not in iodine-rich areas (38). No association with fish intake was reported in a previous EPIC analysis, where very low or very high iodine intakes are rare (39). Intake of grains was not related to TC risk in a meta-analysis of three case-control studies (38). Although anti-cancer effects of olive oil and its compounds are proposed (11), neither olive oil or its compounds were associated with differentiated TC risk in previous EPIC analyses (28, 40). Similar to our findings, the incidence of TC was not related to either dairy products or calcium intake in the NIH-AARP Diet and Health Study, a large US cohort (41). Finally, our results on alcohol are broadly in agreement with a previous EPIC analysis (24) and a meta-analysis of observational studies (42), where moderate alcohol intake was associated with lower risk of TC.

In the current study, low consumption of meat was associated with a 19% lower risk of differentiated TC compared with high consumption. Only a few studies have assessed the direct role of meat intake in TC risk (17). Some classes of meat such as poultry, lamb and pork were positively associated with TC risk in case-control studies conducted in Kuwait (43), Greece (33), and the US (44), but not in Sweden and Norway (45). Potential underlying mechanisms may be related to the concentrations of haem iron and the formation of N-nitroso compounds in meats, especially in red and processed meats (46). Indeed, nitrate can inhibit iodine uptake by the thyroid (17), dysregulate thyroid hormone production and result in thyroid tumor onset (47). Another potential mechanism of action could be the formation of heterocyclic amines and polycyclic aromatic hydrocarbons, which are well-known human mutagens/carcinogens (48). Therefore, the role of meat consumption in thyroid carcinogenesis merits further investigation.

Several limitations of this study should be considered. Dietary data derived from self-reported information relying on participants' memory is prone to measurement error. Dietary data were measured only at recruitment and do not reflect longitudinal changes in dietary intake. Nevertheless, an influence of dietary changes during the pre-diagnostic period of TC is unlikely, since sensitivity analyses excluding incident cases diagnosed within the first 2 years of follow-up were similar to the entire follow-up. Both arMED and rMED scores also have limitations, as a similar weight is given to each component and the foods within them, but not all may have equivalent effects on health or TC risk. Our risk estimates were adjusted for several confounding factors; however, we cannot rule out the possibility of residual confounding by other unmeasured factors. For instance, medical history of benign thyroid diseases, a well-established risk factor for TC was not available in EPIC. The strengths of our study are its prospective design, the relatively large number of TC cases (except for follicular TC subtype), and the wide variation in MD adherence, allowing sufficient statistical power for subgroup analyses. We also minimized any potential bias due to overdiagnosis by stratifying the analysis into countries with high or low incidence rates and into associations with low- or high-risk TC at diagnosis.

## Conclusions

In summary, our study showed no association between adherence to arMED score and differentiated TC risk. However, a potential inverse trend with rMED was suggested, potentially driven by the consumption of a low amount of meat and a moderate amount of alcohol. Future research is required to confirm this potential association with meat intake and to evaluate which type of meat (i.e., red meat, processed meat, poultry, etc.) is responsible for these suggested harmful effects. Lastly, replication and meta-analysis of our findings with other prospective studies is required to further elucidate a possible association with MD adherence.

## Data availability statement

The raw data supporting the conclusions of this article will be made available by the authors, without undue reservation. For information on how to submit an application for gaining access to the EPIC data and/or biospecimens, please follow the instructions at http://epic.iarc.fr/access/index.php.

## Ethics statement

The studies involving human participants were reviewed and approved by Ethical review board of the International Agency for Research on Cancer (IARC). The patients/participants provided their written informed consent to participate in this study.

## Author contributions

RZ-R: conceptualization. AA, MSa, AE, AT, M-CB-R, NL, TT, CLe, VKa, MSc, DP, VKr, SS, RT, FR, GS, TJ, SC, CLa, MR-B, PA, JHu, MG, MA, LN, JH, KP, AH, EW, and SR: data resources. VC: statistical analysis. RZ-R: funding acquisition. FL and MF: writing—original draft preparation. SR and RZ-R: writing—review and editing. All authors have read and agreed to the final version of the manuscript.

## Funding

This research was funded by the Instituto de Salud Carlos III through the grant CP15/00100 (Co-funded by European Regional Development Fund. ERDF, a way to build Europe). The coordination of EPIC is financially supported by the International Agency for Research on Cancer (IARC) and also by the Department of Epidemiology and Biostatistics, School of Public Health, Imperial College London, which has additional infrastructure support provided by the NIHR Imperial Biomedical Research Center (BRC). The national cohorts are supported by: Danish Cancer Society (Denmark); Ligue Contre le Cancer, Institut Gustave Roussy, Mutuelle Générale de l'Education Nationale, Institut National de la Santé et de la Recherche Médicale (INSERM) (France); German Cancer Aid, German Cancer Research Center (DKFZ), German Institute of Human Nutrition Potsdam-Rehbruecke (DifE), Federal Ministry of Education and Research (BMBF) (Germany); Associazione Italiana per la Ricerca sul Cancro-AIRC-Italy, Compagnia di San Paolo and National Research Council (Italy); Dutch Ministry of Public Health, Welfare and Sports (VWS), Netherlands Cancer Registry (NKR), LK Research Funds, Dutch Prevention Funds, Dutch ZON (Zorg Onderzoek Nederland), World Cancer Research Fund (WCRF), Statistics Netherlands (The Netherlands); Health Research Fund (FIS) – Instituto de Salud Carlos III (ISCIII), Regional Governments of Andalucía, Asturias, Basque Country, Murcia and Navarra, and the Catalan Institute of Oncology – ICO (Spain); Swedish Cancer Society, Swedish Research Council and County Councils of Skåne and Västerbotten (Sweden); Cancer Research UK (14136 to EPIC-Norfolk; C8221/A29017 to EPIC-Oxford), Medical Research Council (1000143 to EPIC-Norfolk; MR/M012190/1 to EPIC-Oxford) (United Kingdom). We thank the CERCA Program/Generalitat de Catalunya for the institutional support to Bellvitge Biomedical Research Institute (IDIBELL). RZ-R. was supported by the Miguel Servet program (CPII20/00009) from the Institute of Health Carlos III [Co-funded by the European Social Fund (ESF) – ESF investing in your future].

## Conflict of interest

The authors declare that the research was conducted in the absence of any commercial or financial relationships that could be construed as a potential conflict of interest.

## Publisher's note

All claims expressed in this article are solely those of the authors and do not necessarily represent those of their affiliated organizations, or those of the publisher, the editors and the reviewers. Any product that may be evaluated in this article, or claim that may be made by its manufacturer, is not guaranteed or endorsed by the publisher.

## Author disclaimer

Where authors are identified as personnel of the International Agency for Research on Cancer/World Health Organization, the authors alone are responsible for the views expressed in this article and they do not necessarily represent the decisions, policy or views of the International Agency for Research on Cancer/World Health Organization.

## Abbreviations

arMED: adapted relative Mediterranean diet score
BMI: body mass index
EPIC: European Prospective Investigation into Cancer and Nutrition
MD: Mediterranean diet
rMED: relative Mediterranean diet score
TC: thyroid cancer
T3: triiodothyronine
T4: thyroxine.

## References

[1] Pizzato M Li M Vignat J Laversanne M Singh D La Vecchia C. The epidemiological landscape of thyroid cancer worldwide: GLOBOCAN estimates for incidence and mortality rates in 2020. Lancet Diabetes Endocrinol. (2022) 10:264–72. 10.1016/S2213-8587(22)00035-3 35271818

[2] Vaccarella S Franceschi S Bray F Wild CP Plummer M Dal Maso L. Worldwide thyroid-cancer epidemic? The increasing impact of overdiagnosis. N Engl J Med. (2016) 375:614–7. 10.1056/NEJMp1604412 27532827

[3] Nabhan F Dedhia PH Ringel MD. Thyroid cancer, recent advances in diagnosis and therapy. Int J Cancer. (2021) 149:984–92. 10.1002/ijc.33690 34013533

[4] D'Avanzo B La Vecchia C Franceschi S Negri E Talamini R. History of thyroid diseases and subsequent thyroid cancer risk. Cancer Epidemiol Prev Biomarkers. (1995) 4:193–9. 7606193

[5] Cardis E Kesminiene A Ivanov V Malakhova I Shibata Y Khrouch V. Risk of thyroid cancer after exposure to 131I in childhood. J Natl Cancer Inst. (2005) 97:724–32. 10.1093/jnci/dji129 15900042

[6] Kitahara CM McCullough ML Franceschi S Rinaldi S Wolk A Neta G. Anthropometric factors and thyroid cancer risk by histological subtype: pooled analysis of 22 prospective studies. Thyroid. (2016) 26:306–18. 10.1089/thy.2015.0319 26756356 PMC4754509

[7] Kitahara CM Pfeiffer RM Sosa JA Shiels MS. Impact of overweight and obesity on US papillary thyroid cancer incidence trends (1995–2015). JNCI J Natl Cancer Inst. (2020) 112:810. 10.1093/jnci/djz202 31638139 PMC7825478

[8] Willett WC Sacks F Trichopoulou A Drescher G Ferro-Luzzi A Helsing E T. Mediterranean diet pyramid: a cultural model for healthy eating. Am J Clin Nutr. (1995) 61 (6 Suppl):1402S−6S. 10.1093/ajcn/61.6.1402S 7754995

[9] Bach A Serra-Majem L Carrasco JL Roman B Ngo J Bertomeu I. The use of indexes evaluating the adherence to the mediterranean diet in epidemiological studies: a review. Public Health Nutr. (2006) 9:132–46. 10.1079/PHN2005936 16512961

[10] Buckland G Travier N Cottet V González CA Luján-Barroso L Agudo A. Adherence to the editerranean diet and risk of breast cancer in the European prospective investigation into cancer and nutrition cohort study. Int J Cancer. (2013) 132:2918–27. 10.1002/ijc.27958 23180513

[11] Morze J Danielewicz A Przybyłowicz K Zeng H Hoffmann G Schwingshackl L. An updated systematic review and meta-analysis on adherence to Mediterranean diet and risk of cancer. Eur J Nutr. (2021) 60:1561–86. 10.1007/s00394-020-02346-6 32770356 PMC7987633

[12] Esposito K Kastorini CM Panagiotakos DB Giugliano D. Mediterranean diet and weight loss: meta-analysis of randomized controlled trials. Metab Syndr Relat Disord. (2011) 9:1–12. 10.1089/met.2010.0031 20973675

[13] Esposito K Maiorino MI Bellastella G Chiodini P Panagiotakos D Giugliano D. A journey into a Mediterranean diet and type 2 diabetes: a systematic review with meta-analyses. BMJ Open. (2015) 5:e008222. 10.1136/bmjopen-2015-008222 26260349 PMC4538272

[14] Li H Qian J. Association of diabetes mellitus with thyroid cancer risk: a meta-analysis of cohort studies. Medicine. (2017) 96:e8230. 10.1097/MD.0000000000008230 29381912 PMC5708911

[15] Yeo Y Ma SH Hwang Y Horn-Ross PL Hsing A Lee KE. Diabetes mellitus and risk of thyroid cancer: a meta-analysis. PLoS ONE. (2014) 9:e98135. 10.1371/journal.pone.0098135 24927125 PMC4057085

[16] Mentella MC Scaldaferri F Ricci C Gasbarrini A Miggiano GAD. Cancer and mediterranean diet: a review. Nutrients. (2019) 11:2059. 10.3390/nu11092059 31480794 PMC6770822

[17] Choi WJ Kim J. Dietary factors and the risk of thyroid cancer: a review. Clin Nutr Res. (2014) 3:75. 10.7762/cnr.2014.3.2.75 25136535 PMC4135245

[18] Barrea L Gallo M Ruggeri RM Giacinto PD Sesti F Prinzi N. Nutritional status and follicular-derived thyroid cancer: an update. Crit Rev Food Sci Nutr. (2021) 61:25–59. 10.1080/10408398.2020.1714542 31997660

[19] Hu FB. Dietary pattern analysis: a new direction in nutritional epidemiology. Curr Opin Lipidol. (2002) 13:3–9. 10.1097/00041433-200202000-00002 11790957

[20] Riboli E Hunt K Slimani N Ferrari P Norat T Fahey M. European prospective investigation into cancer and nutrition (EPIC): study populations and data collection. Public Health Nutr. (2002) 5:1113–24. 10.1079/PHN2002394 12639222

[21] Slimani N Deharveng G Unwin I Southgate DA Vignat J Skeie G. The EPIC nutrient database project (ENDB): a first attempt to standardize nutrient databases across the 10 European countries participating in the EPIC study. Eur J Clin Nutr. (2007) 61:1037–56. 10.1038/sj.ejcn.1602679 17375121

[22] Buckland G Agudo A Luján L Jakszyn P Bueno-de-Mesquita HB Palli D. Adherence to a Mediterranean diet and risk of gastric adenocarcinoma within the European prospective investigation into cancer and nutrition (EPIC) cohort study. Am J Clin Nutr. (2010) 91:381–90. 10.3945/ajcn.2009.28209 20007304

[23] Trichopoulou A Costacou T Bamia C Trichopoulos D. Adherence to a Mediterranean diet and survival in a greek population. N Engl J Med. (2003) 348:2599–608. 10.1056/NEJMoa025039 12826634

[24] Sen A Tsilidis KK Allen NE Rinaldi S Appleby PN Almquist M. Baseline and lifetime alcohol consumption and risk of differentiated thyroid carcinoma in the EPIC study. Br J Cancer. (2015) 113:840–7. 10.1038/bjc.2015.280 26313664 PMC4559837

[25] Romaguera D Guevara M Norat T Langenberg C Forouhi NG Sharp S. Mediterranean diet and type 2 diabetes risk in the European prospective investigation into cancer and nutrition (EPIC) study: the InterAct project. Diabetes Care. (2011) 34:1913–8. 10.2337/dc11-0891 21788627 PMC3161259

[26] Rinaldi S Lise M Clavel-Chapelon F Boutron-Ruault MC Guillas G Overvad K. Body size and risk of differentiated thyroid carcinomas: findings from the EPIC study. Int J Cancer. (2012) 131:E1004–14. 10.1002/ijc.27601 22511178

[27] Zamora-Ros R Rinaldi S Biessy C Tjønneland A Halkjaer J Fournier A. Reproductive and menstrual factors and risk of differentiated thyroid carcinoma: the EPIC study. Int J Cancer. (2015) 5:1218–27. 10.1002/ijc.29067 25041790

[28] Zamora-Ros R Rinaldi S Tsilidis KK Weiderpass E Boutron-Ruault MC Rostgaard-Hansen AL. Energy and macronutrient intake and risk of differentiated thyroid carcinoma in the European prospective investigation into cancer and nutrition study. Int J Cancer. (2016) 138:65–73. 10.1002/ijc.29693 26190646 PMC6300115

[29] Wareham NJ Jakes RW Rennie KL Schuit J Mitchell J Hennings S. Validity and repeatability of a simple index derived from the short physical activity questionnaire used in the European prospective investigation into cancer and nutrition (EPIC) study. Public Health Nutr. (2003) 6:407–13. 10.1079/PHN2002439 12795830

[30] Agudo A Slimani N Ocké MC Naska A Miller AB Kroke A. Consumption of vegetables, fruit and other plant foods in the European prospective investigation into cancer and nutrition (EPIC) cohorts from 10 European countries. Public Health Nutr. (2002) 5:1179–96. 10.1079/PHN2002398 12639226

[31] Liang J Zhao N Zhu C Ni X Ko J Huang H. Dietary patterns and thyroid cancer risk: a population-based case-control study. Am J Transl Res. (2020) 12:180–90. 32051747 PMC7013212

[32] Cléro É Doyon F Chungue V Rachédi F Boissin JL Sebbag J. Dietary patterns, goitrogenic food, and thyroid cancer: a case-control study in French polynesia. Nutr Cancer. (2012) 64:929–36. 10.1080/01635581.2012.713538 23061901

[33] Markaki I Linos D Linos A. The influence of dietary patterns on the development of thyroid cancer. Eur J Cancer. (2003) 39:1912–9. 10.1016/s0959-8049(03)00432-5 12932671

[34] Zupo R Castellana F Panza F Lampignano L Murro I Di Noia C. Adherence to a mediterranean diet and thyroid function in obesity: a cross-sectional apulian survey. Nutrients. (2020) 12:1–10. 10.3390/nu12103173 33081337 PMC7603040

[35] Sasson M Kay-Rivest E Shoukrun R Florea A Hier M Forest VI. The T4/T3 quotient as a risk factor for differentiated thyroid cancer: a case control study. J Otolaryngol Head Neck Surg. (2017) 46:28. 10.1186/s40463-017-0208-0 28376913 PMC5379683

[36] Rinaldi S Plummer M Biessy C Tsilidis KK Østergaard JN Overvad K. Thyroid-stimulating hormone, thyroglobulin, and thyroid hormones and risk of differentiated thyroid carcinoma: the EPIC study. J Natl Cancer Inst. (2014) 106:dju097. 10.1093/jnci/dju097 24824312

[37] Zamora-Ros R Béraud V Franceschi S Cayssials V Tsilidis KK Boutron-Ruault MC. Consumption of fruits, vegetables and fruit juices and differentiated thyroid carcinoma risk in the European prospective investigation into cancer and nutrition (EPIC) study. Int J Cancer. (2018) 142:449–59. 10.1002/ijc.30880 28688112 PMC6198931

[38] Liu ZT Lin AH. Dietary factors and thyroid cancer risk: a meta-analysis of observational studies. Nutr Cancer. (2014) 66:1165–78. 10.1080/01635581.2014.951734 25256273

[39] Zamora-Ros R Castañeda J Rinaldi S Cayssials V Slimani N Weiderpass E. Consumption of fish is not associated with risk of differentiated thyroid carcinoma in the European prospective investigation into cancer and nutrition (EPIC) study. J Nutr. (2017) 147:1366–73. 10.3945/jn.117.247874 28592517

[40] Zamora-Ros R Cayssials V Franceschi S Kyrø C Weiderpass E Hennings J. Polyphenol intake and differentiated thyroid cancer risk in the European prospective investigation into cancer and nutrition (EPIC) cohort. Int J Cancer. (2020) 146:1841–50. 10.1002/ijc.32589 31342519

[41] Park Y Leitzmann MF Subar AF Hollenbeck A Schatzkin A. Dairy food, calcium, and risk of cancer in the NIH-AARP diet and health study. Arch Intern Med. (2009) 169:391–401. 10.1001/archinternmed.2008.578 19237724 PMC2796799

[42] Hong SH Myung SK Kim HS. Alcohol intake and risk of thyroid cancer: a meta-analysis of observational studies. Cancer Res Treat. (2017) 49:534–47. 10.4143/crt.2016.161 27456949 PMC5398382

[43] Memon A Varghese A Suresh A. Benign thyroid disease and dietary factors in thyroid cancer: a case-control study in Kuwait. Br J Cancer. (2002) 86:1745–50. 10.1038/sj.bjc.6600303 12087461 PMC2375394

[44] Daniel CR Cross AJ Graubard BI Hollenbeck AR Park Y Sinha R. Prospective investigation of poultry and fish intake in relation to cancer risk. Cancer Prev Res. (2011) 4:1903–11. 10.1158/1940-6207.CAPR-11-0241 21803982 PMC3208759

[45] Galanti MR Hansson L Bergström R Wolk A Hjartåker A Lund E. Diet and the risk of papillary and follicular thyroid carcinoma: a population-based case-control study in Sweden and Norway. Cancer Causes Control. (1997) 8:205–14. 10.1023/a:1018424430711 9134245

[46] Cross AJ Pollock JRA Bingham SA. Haem, not protein or inorganic iron, is responsible for endogenous intestinal N-nitrosation arising from red meat. Cancer Res. (2003) 63:2358–60. 12750250

[47] Aschebrook-Kilfoy B Shu X-O Gao Y-T Ji BT Yang G Li HL. Thyroid cancer risk and dietary nitrate and nitrite intake in the Shanghai Women's health study. Int J Cancer. (2013) 132:897–904. 10.1002/ijc.27659 22674227 PMC3443521

[48] Cross AJ Sinha R. Meat-related mutagens/carcinogens in the etiology of colorectal cancer. Environ Mol Mutagen. (2004) 44:44–55. 10.1002/em.20030 15199546

