# Supplementary material for: Adherence to mediterranean diet and the risk of differentiated thyroid cancer in a European cohort: The EPIC study
Source: Front Nutr. 2022 Sep 2;9:982369. doi: 10.3389/fnut.2022.982369 (PMC9481277; doi:10.3389/fnut.2022.982369)
Supplement: Supplementary file 1 [file Data_Sheet_1.docx]

Supplementary Material

**Supplementary Table 1.** Subgroup and sensitivity analyses of the associations between adapted relative Mediterranean diet score (arMED) and differentiated thyroid cancer (TC) risk in the EPIC study.

|  | | **N** | **Cases (n)** | **arMED score** | | | | |
| --- | --- | --- | --- | --- | --- | --- | --- | --- |
|  |  |  |  | **Low (0-5)** | **Medium (6-9)** | **High (10-16)** | **p-trend** | **Continuous (per unit arMED)** |
|  |  |  |  | **HR (95% CI)** | **HR (95% CI)** | **HR (95% CI)** |  | **HR (95% CI)** |
| Women | | 318,647 | 638 | 1.00 (ref) | 1.08 (0.82-1.41) | 0.88 (0.65-1.19) | 0.14 | 0.98 (0.94-1.01) |
| Disease stage | |  |  |  |  |  |  |  |
|  | Low risk (T1-T2) | 450,064 | 371 | 1.00 (ref) | 1.10 (0.79-1.55) | 0.82 (0.56-1.21) | 0.10 | 0.97 (0.92-1.01) |
|  | High risk (T3-T4) | 450,064 | 97 | 1.00 (ref) | 1.75 (0.81-3.75) | 1.47 (0.63-3.42) | 0.76 | 1.01 (0.92-1.11) |
| TC incidence rate^2^ | |  |  |  |  |  |  |  |
|  | Low | 249,595 | 175 | 1.00 (ref) | 1.07 (0.73-1.57) | 1.08 (0.63-1.85) | 0.75 | 1.00 (0.93-1.08) |
|  | High | 200,469 | 537 | 1.00 (ref) | 1.15 (0.82-1.60) | 0.92 (0.64-1.32) | 0.14 | 0.98 (0.94-1.02) |
| EPIC regions^3^ | |  |  |  |  |  |  |  |
|  | North Europe | 137,643 | 114 | 1.00 (ref) | 0.98 (0.62-1.54) | 1.64 (0.88-3.06) | 0.22 | 1.05 (0.97-1.15) |
|  | Central Europe | 160,503 | 143 | 1.00 (ref) | 1.09 (0.73-1.63) | 0.63 (0.33-1.20) | 0.31 | 0.97 (0.89-1.05) |
|  | South Europe | 151,918 | 455 | 1.00 (ref) | 1.33 (0.81-2.19) | 1.08 (0.65-1.79) | 0.18 | 0.97 (0.93-1.01) |
| Excluding TC cases <2 y of follow-up | | 449,988 | 636 | 1.00 (ref) | 1.08 (0.83-1.41) | 0.89 (0.66 1.20) | 0.17 | 0.97 (0.94-1.01) |

^1^Cox models were stratified by sex, age at recruitment, study center, and adjusted for total energy intake (kcal/day, continuous), body mass index (continuous), smoking status, alcohol (g/day, continuous), education level, and physical activity. In addition, in women, the model was further adjusted for menopausal status and type, ever use of oral contraceptives, and history of infertility problems.

^2^EPIC countries by differentiated thyroid cancer incidence rates (>5/10,000 in women): High (France, Germany, Italy and Spain), and Low (Denmark, the Netherlands, Norway, Sweden and UK).

**Supplementary Figure 1:** Participant flowchart of the EPIC study

**N= 521,324 (857 TC)**

**29332 (45 Thyroid cancer (TC) event)**

25184 (45 TC) Prevalent cancer cases

4148 Date of diagnosis or check is missing, or length of follow-up is zero

**N= 491,992 (812 TC)**

**1329 (52 TC)** Causes of exclusion:

1277 No lifestyle information (i.e. did not complete the questionnaire)

52 TC due to classification: Other specified morphology = 5

Medullary = 37

Anaplastic = 9

Lymphoma = 1

**N= 490,663 (760 TC)**

**14555** **(12 TC)** Causes of exclusion:

4982 (3 TC) No dietary or lifestyle information (i.e. did not complete the questionnaire)

9573 (9 TC) Extreme ranking on the ratio energy intake/energy requirement (top and bottom 1%).

**N=476,108 (748 TC)**

**26044** **(36 TC)** Participants from Greece excluded.

**N=450,064**

**(712 TC)**
